# Do East Asians With Normal Glucose Tolerance Have Worse β-Cell Function? A Meta-Analysis of Epidemiological Studies

**DOI:** 10.3389/fendo.2021.780557

**Published:** 2021-11-30

**Authors:** Li Li, Xiantong Zou, Qi Huang, Xueyao Han, Xianghai Zhou, Linong Ji

**Affiliations:** Department of Endocrinology and Metabolism, Peking University People’s Hospital, Beijing, China

**Keywords:** diabetes, β-cell function, ethical differences, insulin resistance, prediabetes

## Abstract

**Background:**

The difference in the relationship between β-cell function and insulin resistance among Africans, Caucasians and East Asians with normal glucose tolerance (NGT) was not well investigated.

**Methods:**

We searched PubMed and Web of Science with keywords and identified studies that used the homeostasis model assessment (HOMA) model to evaluate β-cell function (HOMA-B) and insulin sensitivity/resistance (HOMA-S/HOMA-IR) in certain ethnic groups. We used random-effect model to pool data of HOMAs and compared the combined data among the three ethnic groups using subgroup analysis. Linear regression analysis was used to estimate the coefficient of HOMA-S on HOMA-B in these ethnic groups.

**Results:**

We evaluated pooled data of HOMAs in eight African, 26 Caucasian, and 84 East Asian cohorts with NGT, and also 2,392, 6,645 and 67,317 individuals, respectively. The three ethnic groups had distinct HOMA-B but similar HOMA-IR. The regression coefficient of lnHOMA-B on lnHOMA-S was different between Africans and Caucasians (−1.126 *vs* −0.401, P = 0.0006) or East Asian (−1.126 *vs* −0.586, P = 0.0087), but similar between Caucasians and East Asians (−0.401 *vs* −0.586, P = 0.1282). The coefficient in all ethnic groups was similar when age, BMI, and gender were adjusted (African *vs* Caucasian P = 0.0885, African *vs* East Asian P = 0.1092, and Caucasian *vs* East Asian P = 0.6298).

**Conclusions:**

In subjects with NGT, East Asians had lower HOMA-B but similar β-cell response relative to insulin resistance with Caucasians and Africans when age, BMI, and gender were controlled. This result may challenge the allegation that there was an Asian-specific diabetes phenotype with worse β-cell function.

## Introduction

The latest atlas of the International Diabetes Federation (IDF) indicated that there were 162.6 million people with diabetes in the Western Pacific Region in 2019, which was 35% of the world’s total number of adults with diabetes, ranking the first among all regions (1). ‘Asian type diabetes’ was proposed since Asian people with diabetes had earlier age-of-onset, lower BMI, and some unique susceptible genetic loci related to diabetes (2, 3) compared with non-Asian counterparts. Studies suggested that East Asians, either with or without diabetes, had poor β-cell function than other ethnicities, either assessed by oral glucose tolerance test (OGTT) or by frequently sampled intravenous glucose tolerance tests (FSIGT) (4–7). Compared with Caucasians, Asian subjects with normal glucose tolerance (NGT), pre-diabetes or diabetes all had lower insulin secretion post 75-g glucose challenge (8). Nevertheless, despite ‘worse’ β-cell function, East Asians are less resistant to insulin (6) and have relatively lower BMI (9). It was well established that the relationship between β-cell function and insulin sensitivity was linked through a negative feedback loop (10–12). Bergman et al. first proposed a hyperbolic function could characterize this relationship: the product of β-cell response and insulin sensitivity was a constant (13), and Kahn et al. verified it by using regression analysis with log transformation: log (insulin secretion) = constant − log (insulin sensitivity) (14). An ideal equation may have a coefficient of log (insulin sensitivity) close to −1 (11, 14). The β-cell function in East Asians may be underestimated without considering their insulin sensitivity.

Whether East Asians had poor insulin secretion response to insulin resistance was largely unknown until Kodama et al. pooled the data of FSIGT to evaluate the relationship between insulin sensitivity and β-cell function in different ethnicities (7). They reported ethnic differences in the optimal points in the canalization of normal blood glucose levels, and speculated that East Asians were more susceptible to diabetes, and a small change in insulin resistance may lead to drastic variations in their β-cell function (7). However, the studies referenced by Kodama et al. were all FISIGT studies with small sample sizes, especially in East Asians, possibly due to the inconvenience of sampling. This study aimed to investigate the ethical difference in the relationship between β-cell function and insulin resistance with the homeostasis assessment (HOMA) model calculated from fasting glucose and insulin levels (15) in subjects with NGT in large-scale epidemiological studies.

## Methods

The Preferred Reporting Items for Systematic reviews and Meta-Analyses (PRISMA) was used as guidance for our meta-analysis (16).

### Study Selection

In this meta-analysis, studies met the following inclusion criteria: 1) age of study subjects ≥18 years; 2) β-cell function and insulin resistance (or insulin sensitivity) were measured by HOMA method (15) or HOMA-2 calculator provided on the Oxford website (https://www.dtu.ox.ac.uk/homacalculator/download.php); 3) data of HOMAs can be extracted by different ethnic groups in participants with normal glucose tolerance (NGT). Studies were excluded when subjects had other diseases influencing glycemic control or anti-glycemic therapy, baseline information was unavailable, or HOMAs were miscalculated using wrong formula.

### Data Sources and Searches

We searched PubMed for studies that used the HOMA model to measure β-cell function and insulin resistance (or insulin sensitivity) before February 2020. The strategy was performed using the following terms: [“HOMA-β” AND (“HOMA-IS” OR “HOMA-IR” OR “Caucasian” OR “African” OR “Chinese” OR “Japanese” OR “Korean”)] OR [“β-cell function” AND (“HOMA-IS” OR “HOMA-IR” OR “Caucasian” OR “African” OR “Chinese” OR “Japanese” OR “Korean”)]. We also reviewed the reference lists and supplementary material of eligible publications and manually searched literature on Web of Science (https://apps-webofknowledge-com./). The language of the articles was restricted to English. We contacted original authors by sending e-mails if necessary. Two review authors independently extracted data from each publication, and disagreements or discrepancies were resolved by discussion.

### Data Extraction and Quality Assessment

We extracted the following data from each publication using a unified form: publication information (first author, year and PMID); sample size of the study population; study subjects’ mean age, BMI, HOMA-B, and HOMA-IR (or HOMA-S, which is the reciprocal of HOMA-IR). We chose the most detailed study from its duplicates.

We used the modified Newcastle-Ottawa Scale (MNOS), which was developed to assess the quality of non-randomized studies in meta-analysis (http://www.ohri.ca/programs/clinical_epidemiology/oxford.asp), to assess the quality of included studies based on three items: study selection (e.g., representativeness of the cohort and purity of ethnic groups), confounding factors and measurement of HOMAs. We grade all studies and defined studies obtaining eight or nine points as high quality, seven points as medium, and ≤six points as poor on a scale of zero to nine points (7).

### Data Synthesis and Analysis

To unify data types of presented HOMA values, we transformed median- (range interquartile) into meta-analytic mean-(standard deviation) using methods proposed by Wan et al. (17) and Luo et al. (18) (both available on http://www.math.hkbu.edu.hk/~tongt/papers/median2mean.html). We pooled mean values of HOMA-B, HOMA-IR (or HOMA-IS), BMI, and age in three ethnic groups using the random-effect model (19). Subgroup analyses were performed to pool and compare data of HOMAs among different ethnic groups by random-effect model, and sensitivity analysis was performed to assess the robustness of results (20). The heterogeneity among studies was estimated by I^2^ statistics (21). Results were presented as mean (95% CI). HOMA-B and HOMA-S were plotted against each other, and the hyperbolic curve was fitted. Liner regression of ln-transformed HOMAs was used to evaluate the relationship between HOMA-B and HOMA-S (14, 22, 23). The regression coefficients among subgroups in crude or adjusted with confounders, e.g., BMI, age and male percentage, were compared using the Wald test. All statistical analysis was performed with the Stata statistical software package (version 16.0), and P < 0.05 was considered to be statistically significant.

## Results

**Figure 1:**
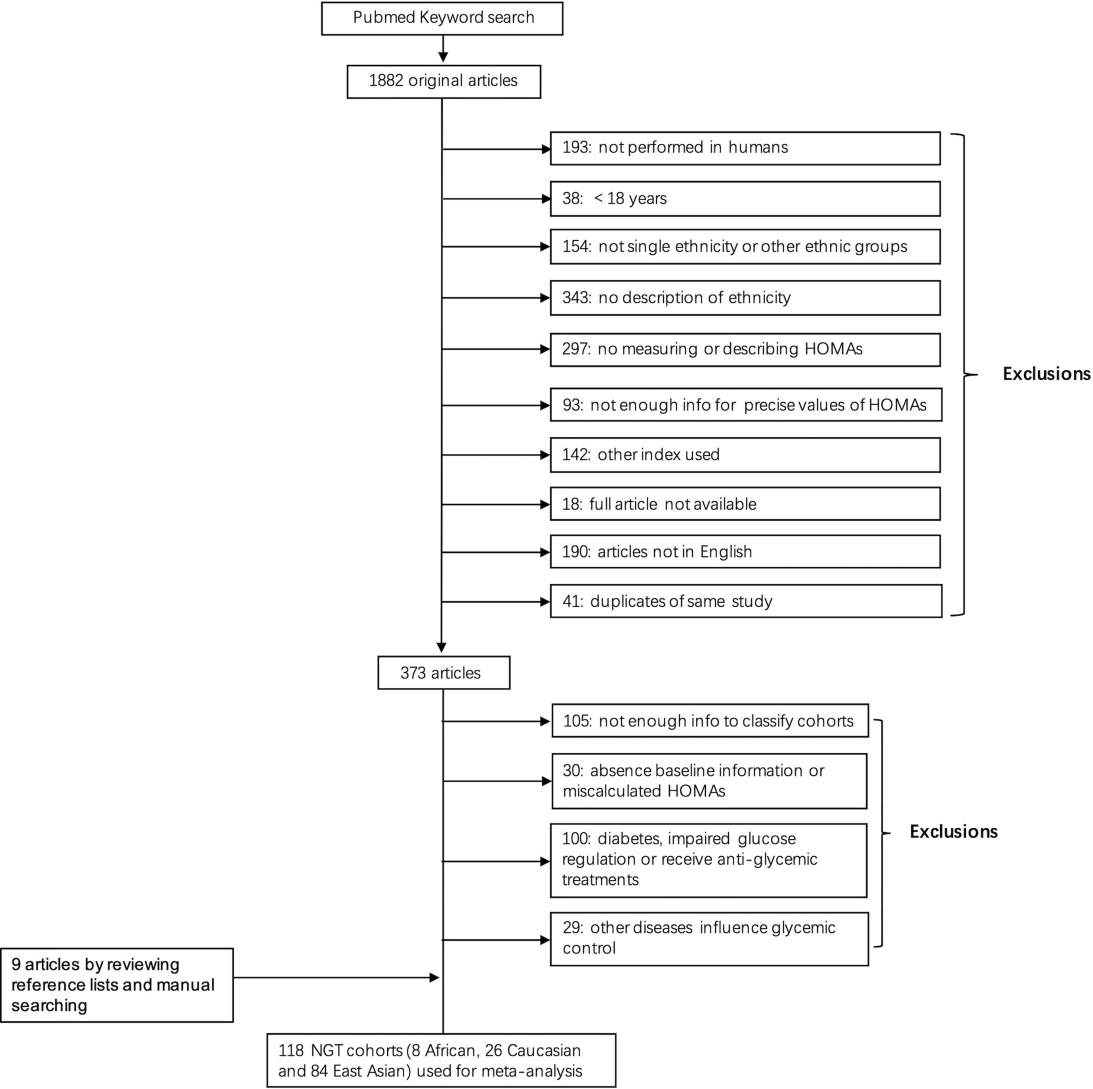
Flow diagram of literature search and cohort identification.

We searched on PubMed using keywords and obtained 1,882 original articles, of which 1,509 were excluded because of study subjects’ characteristics (e.g., age <18 or from other ethnicities), inadequate HOMA analysis (e.g., only measuring HOMA-B), and other situations (e.g., duplication study or full article unavailable). We reviewed the ones left and further excluded studies: 1) study subjects had unclassified glucose tolerance, 2) study subjects had other diseases or took medications that influence glycemic control, or 3) studies used wrong equation to calculate HOMAs. Eventually, 118 cohorts with normal glucose tolerance containing three ethnic groups were identified: eight African, 26 Caucasian, and 84 East Asian cohorts comprised of 2,392, 6,645, and 67,317 individuals, respectively. The selection process was summarized in **Figure 1**. The MNOS assessment showed that six African cohorts had high quality (maximum nine points), and the other two cohorts had low quality (obtaining only five or six points); of the 26 Caucasian cohorts, 22 had high quality (eight cohorts got nine points and 14 got eight points) and the other four cohorts had medium quality; of the 84 East Asian cohorts, 74 had high quality (34 got nine points and 40 got eight points), nine had medium quality and the left one had low quality (six points).

Mean age, BMI, HOMA-B, and HOMA-IR were pooled and compared among the three ethnic groups. East Asians were older with lower BMI than the other two groups. Caucasians had higher HOMA-B than Asians, as indicated by the 95% CI (**Table 1**). Subgroup analysis showed that three ethnic groups had significance in HOMA-B (P = 0.0008) but not in HOMA-IR (P = 0.1770) (**Table 1** and **Supplementary Figure S1**).

**Table 1:** Ethnic characteristics of pooled data in NGT population.

	African	Caucasian	East Asian
N (% male)	2173 (19.10)	5778 (30.02)	64368 (48.34)
Age (years)*	40.19 (36.80, 43.58)	38.25 (35.22, 41.27)	47.01 (43.62, 50.41)
BMI (kg/m^2^)*	28.78 (26.80, 30.75)	26.11 (25.13, 27.10)	23.60 (23.39, 23.81)
HOMA-IR	2.06 (1.35, 2.78)	1.94 (1.62, 2.26)	1.67 (1.56, 1.77)
HOMA-B*	175.01 (101.56, 248.46)	130.96 (117.93, 144.00)	103.14 (93.86, 112.42)

Data are means (95% CI). Data were pooled across the three ethnic groups using random-effect model. East Asians had lower BMI than Africans or Caucasians. HOMA-B progressively decreased in Africans, Caucasians and East Asians along with HOMA-IR decreasing accordingly. *P < 0.001 by subgroup analysis. NGT, normal glucose tolerance; HOMA-IR, homeostasis model assessment-insulin resistance; HOMA-B, homeostasis model assessment-β-cell function.

**Figure 2:**
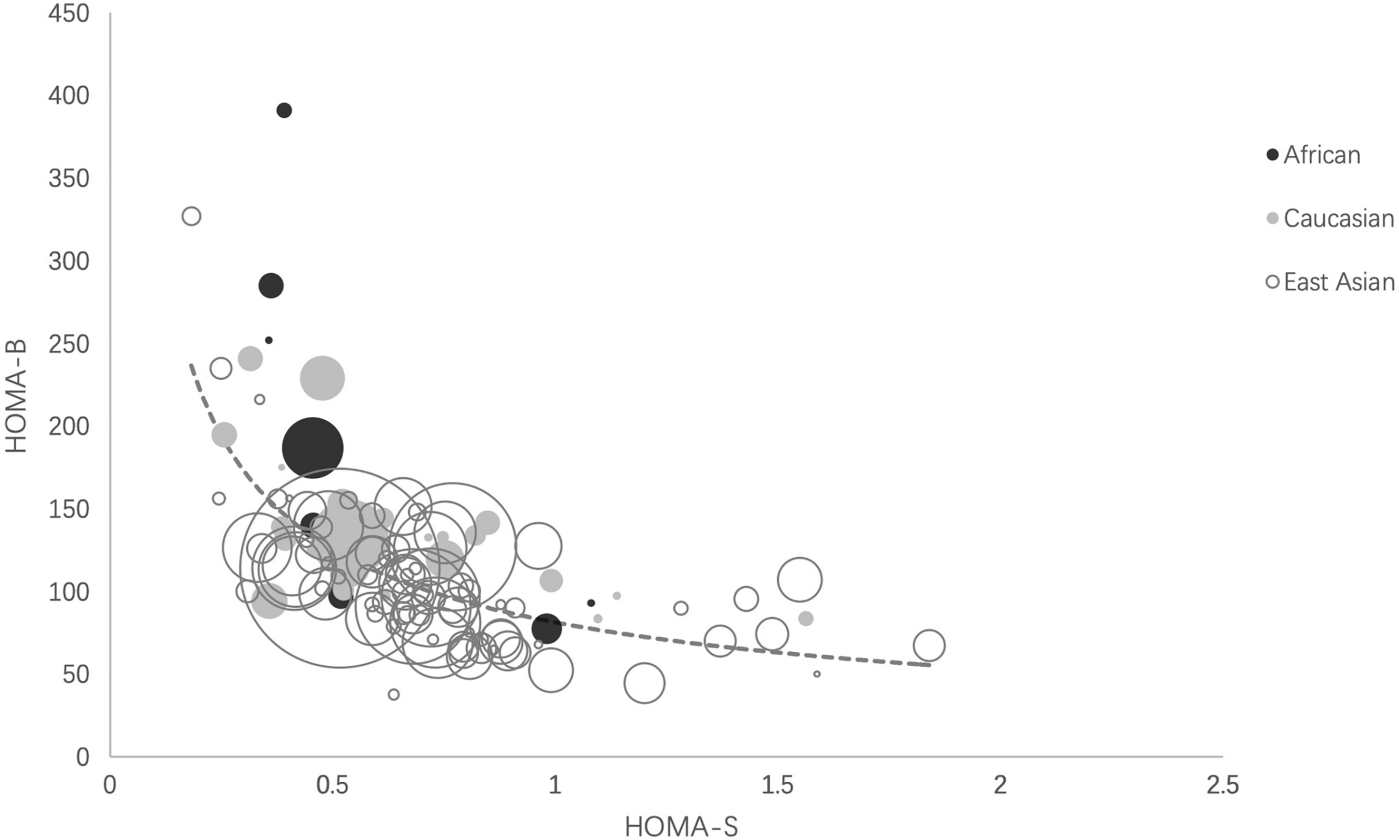
Ethnic distributions of HOMA-B relative to HOMA-S. The scatter plot reflects the hyperbolic relationship between HOMA-B and HOMA-S across African, Caucasian, and East Asian cohorts. Each circle indicates one cohort and the circle area is proportional to sample size of the cohort. The curve was calculated as ln (HOMA-B) = −0.626 ln (HOMA-S) + 4.399 by liner regression analysis. HOMA-S, homeostasis model assessment-insulin sensitivity; HOMA-B, homeostasis model assessment-β-cell function.

We plotted the mean values of HOMA-B against HOMA-S in the NGT (healthy) cohorts in the three ethnicities. We fitted the HOMA-B and HOMA-S in a hyperbolic curve [ln (HOMA-B) = −0.626 ln (HOMA-S) + 4.399]. Most cohorts clustered and located around the center of the hyperbola, especially those with large sample sizes. Some East Asian cohorts with small sample sizes were distributed at both ends and a few African cohorts with small sample sizes were located at the upper end of the curve (**Figure 2**).

To investigate the hyperbolic relationship between HOMA-S and HOMA-B, we ln-transformed these two parameters and calculated the coefficient of lnHOMA-S on lnHOMA-B using a linear regression model. lnHOMA-S and ln HOMA-B were all fitted into a linear regression model before and after adjustment in all ethnicities (p <0.001). In crude, the coefficient in Africans was significantly lower than that in Caucasians or East Asians (**Table 2**), and the coefficients had no significant difference between East Asians and Caucasians. No significant difference was observed among the three ethnic groups after age, BMI and/or gender was adjusted (**Table 2** and **Supplementary Figure S2**).

**Table 2:** Coefficients of lnHOMA-S on lnHOMA-B across the three ethnic groups.

Ethnicity	N	Unadjusted		Adjusted age and BMI		Adjusted age, BMI, and gender	
**African**	2,392	−1.126 (−1.497, −0.754)	P = 0.0006 *vs* Caucasian	−1.176 (−2.010, −0.343)	P = 0.0784 *vs* Caucasian	−1.303 (−2.282, −0.324)	P = 0.0885 *vs* Caucasian
**Caucasian**	6,645	−0.401 (−0.582, −0.219)	P = 0.1282 *vs* East Asian	−0.409 (−0.596, −0.223)	P = 0.4827 *vs* East Asian	−0.443 (−0.588, −0.298)	P = 0.6298 *vs* East Asian
**East Asian**	67,317	-0.586 (−0.742, −0.430)	P = 0.0087 *vs* African	−0.493 (−0.636, −0.350)	P = 0.1136 *vs* African	−0.494 (−0.642, −0.346)	P = 0.1092 *vs* African

The relationship between lnHOMA-B and lnHOMA-S was analyzed using liner regression analysis in African, Caucasian and East Asian cohorts. The coefficient presented as mean (95% confidence interval) of the liner function before and after adjustment of age, BMI or gender was presented. HOMA-S, homeostasis model assessment-insulin sensitivity; HOMA-B, homeostasis model assessment-β-cell function.

## Discussion

Our study demonstrated a significant difference in the HOMA-B level but similar HOMA-IR level among East Asian, African, and Caucasian study subjects with NGT. East Asians had significantly lower HOMA-B than Caucasians. The relationship between HOMA-S and HOMA-B was similar across three ethnic groups when BMI, age and/or gender were adjusted.

Our study showed that the pooled data of HOMA-B and HOMA-S of three ethnic groups all clustered and overlapped around the center of the hyperbolic curve, indicating that East Asians had a similar relationship between HOMA-B and HOMA-S with Caucasians and Africans. Similar with our findings, one study reported that β-cell function adjusted by insulin sensitivity in Asians showed no significant difference among the three ethnic groups (6). In contrast, Kodama et al. found that the African cohorts clustered at the upper end and East Asian cohorts clustered at the lower end of the hyperbolic curve and speculated this might contribute to unstable glucose canalization in these ethnic groups (7). The unbalanced distribution of these two ethnicities may be attributable to small sample sizes in East Asian (n = 205) and African cohorts (n = 688) with NGT in that study (7). In addition, the inclusion criteria of our study were different from Kodama et al. They included adolescents and excluded participants with BMI >30 kg/m^2^, whereas we mainly recruited with a wide BMI range. Our study greatly expanded the sample size of all three groups using epidemiological data and the statistical power to test the hypothesis of whether the ethnic difference exists.

As previously demonstrated by other studies, East Asians had the lowest HOMA-B compared with Caucasians and Africans. The insulin resistance showed no significant difference among three ethnic groups. Our hypothesis that the reduced β-cell function was secondary to lower insulin resistance rather than β-cell function impairment *per se* was supported by the findings that the relationship between HOMA-S and HOMA-B was consistent among three ethnic groups after BMI and age were adjusted. This suggested that if East Asians had significantly elevated insulin resistance, as much as the Caucasians, their insulin secretion may compensatively increase in NGT study subjects. Unlike other studies that indicated Asians had less insulin resistance than others (6), our study showed there was a non-significant trend of decrease in the pooled HOMA-IR in Asians. This could be possibly due to the large variance and relatively small sample size in African cohorts. Africans had a high coefficient in the same increment of HOMA-S. However, this was primarily attributable to BMI and age. In this study we adjusted age, BMI and gender since numerous studies have demonstrated that BMI, age and gender may affect the ethnic difference in the relationship between insulin secretion and insulin sensitivity (24–27). In fact, insulin resistance was higher in study subjects with higher BMI (28) and insulin response had no significant difference between East Asians and Caucasians after controlling for BMI (25). Also, age was shown to affect insulin resistance and β-cell function in different ethnic groups with NGT (29–31).

The constant of the hyperbolic curve, which is the product of insulin secretion and insulin sensitivity, is frequently called the disposition index (DI), reflecting β-cell response compensated for insulin resistance (14, 32). We pooled the AIR*Si using IFSGT data from NGT derived from references included by Kodama et al. and references from Web of Science, and our results showed that East Asians had similar DI with other ethnic groups (**Supplementary Figure S3**). Many studies suggested DI may be consistent among ethnicities when confounders affecting glucose homeostasis were adjusted. Studies indicated that after matching or adjusting for BMI and/or age, DI derived from FSIGT or Clamp studies were similar among different ethnic groups (27, 33, 34). In fact, Moller et al. discovered that DI was consistent between Japanese and Caucasians not only in NGT but also in subjects with IGT and type 2 diabetes using oral glucose tolerance test (OGTT) (8). Our data additionally supported that glucose homeostasis’ stabilization point was consistent across different ethnicities as a summary of epidemiological evidence.

It was proposed that Asian diabetes should be categorized as subgroup of diabetes since East Asians had poor β-cell function, higher postprandial glucose excursion, and responded to glucose lowering medication differently (2) and difference response to some anti-diabetes medication (35, 36). Our result suggests that the relationship between β-cell function and insulin resistance may be similar among three ethnic groups. The β-cell function may be underestimated in East Asians due to their high insulin sensitivity. Whether Asians deserve particular glucose-lowering therapy was still in dispute since there was robust evidence the ethnic difference in anti-diabetes therapies may not exist (37–40). Taken together, we propose that there is no sufficient evidence to support ‘Asian type diabetes’ either to emphasize the difference in β-cell function or to promote ethnic-specific treatment. Etiological and pathogenesis studies, including genetic susceptibility, molecular biological mechanism, and clinical trials regarding the difference among ethnic groups should consider that the relative relationship between β-cell function and insulin resistance was similar across ethnic groups.

Another important question regarding the ethnical difference in β cell function was whether post glucose challenge insulin secretion differed among ethnic groups. Our data only measured fasting β-cell function since epidemiological studies mainly solely measured glucose insulin at fasting status. Kodama et al. pooled data from IVGTT and found that the acute insulin response to glucose (AIRg) was lower in Asian subjects than African and Caucasian subjects (7). Chiu et al. found that the second phase insulin secretion of Asians was lower than Mexican Americans but higher than Caucasians and African Americans (27). β-cell response estimated using oral minimal models (OMM) was lower in Japanese surjects at basal but similar at dynamic and static status compared with Caucasians especially when BMI was adjusted (8). Few studies investigated the ethnical difference in the relative relationship between post-challenge insulin secretion and insulin resistance. It was found that the static DI was similar between Japanese and Caucasians regardless of the glucose tolerance status (8). Further studies were necessary to gather more information on post challenge β-cell function in reference to insulin resistance.

Our study had an advantage in sample size by pooling data from large-scale population-based epidemiological surveys in study subjects with NGT with HOMAs since these values were convenient and widely used in many epidemiological studies (41–44). We also adjusted BMI, age, and gender confounders to reveal the underlying association between β-cell function and insulin sensitivity in different ethnic groups. There were also limitations in our studies: (1) HOMA models calculated using fasting glucose and insulin level at one single time were less stable than index derived from IVGTT or OGTT. We were unable to assess insulin secretion post glucose challenge and its relative relationship with insulin sensitivity. However, HOMA models were widely used to investigate the hyperbolic relationship before (22), and estimates derived from HOMA models correlate well with that from hyperglycemic clamps and IVGTT (15, 42). An optimal hyperbolic function required a coefficient close to −1. Our study shows that coefficients in Caucasian and East Asian groups were far from −1, limiting the use of the HOMA model in analyzing the hyperbolic functions (11, 14). (2) Another limitation was the relatively high heterogeneities which may result from the following reasons: 1) research design and qualities of included studies varied across ethnicities; 2) the included cohorts from different studies may not have precisely the same glucose tolerance given that some healthy or control cohorts from volunteer-based studies included study subjects who had unknown impaired glucose tolerance (IGT) or took unreported medications that could influence glycemic control; 3) some cohorts may have impure ethnic ancestry—some of the African cohorts included African-Americans, and some cohorts were based on self-reported ancestry. Despite of this, our sensitivity analysis shows no outlier study cohorts that could affect our combined results (data not shown). (3) Finally, potential selection bias was inevitable. There were a large number of Asian studies but studies from African ancestry were less. We mainly searched PubMed and Web of Science using keywords and restricted language to English and lost a few studies. Despite these limitations, this is the first study to investigate hyperbolic relationships between HOMA-B and HOMA-S across three ethnic groups by comparing the parameters derived from their corresponding hyperbolic functions before or after adjustment for age and BMI.

In conclusion, East Asians had lower β-cell function and similar insulin sensitivity compared with Caucasians and Africans. The relationship between β-cell function and insulin sensitivity was consistent among the three ethnic groups, especially when age and BMI were adjusted. This study challenged the conventional impression that East Asians had poor β-cell functions and the existence of Asian type diabetes, which may give new insights to clinicians in making clinical recommendations in patients from different ethnic background and researchers in implicating etiological studies on the pathogenesis of diabetes in different ethnic groups.

## Data Availability Statement

The original contributions presented in the study are included in the article/**Supplementary Material**. Further inquiries can be directed to the corresponding authors.

## Author Contributions

LL and XTZ did the literature research and created the figures and tables. LL and XTZ did the data collection, data analysis, and writing of the report. LJ contributed to the design of this study and reviewed the manuscript. QH contributed to data analysis. XH and XHZ reviewed the manuscript. All authors contributed to the article and approved the submitted version.

## Funding

Work on this study was supported by a grant from Beijing Nova Program of Science and Technology (Z191100001119026) to XTZ and grants from the National Key R&D Program of China to LJ (2016YFC1304901) and XHZ (2016YFC1305603) and a grant from Peking University People’s Hospital Research And Development Funds (RDX2019-11) to XTZ

## Conflict of Interest

The authors declare that the research was conducted in the absence of any commercial or financial relationships that could be construed as a potential conflict of interest.

## Publisher’s Note

All claims expressed in this article are solely those of the authors and do not necessarily represent those of their affiliated organizations, or those of the publisher, the editors and the reviewers. Any product that may be evaluated in this article, or claim that may be made by its manufacturer, is not guaranteed or endorsed by the publisher.
