# Supplementary material for: Do East Asians With Normal Glucose Tolerance Have Worse β-Cell Function? A Meta-Analysis of Epidemiological Studies
Source: Front Endocrinol (Lausanne). 2021 Nov 30;12:780557. doi: 10.3389/fendo.2021.780557 (PMC8669805; doi:10.3389/fendo.2021.780557)
Supplement: Supplementary file 1 [file DataSheet_1.docx]

**S****upporting Information:**

Figure S1

Figure S1A.

Figure S1B.

Figure S1. Forest plots of HOMA-IR (A) and HOMA-B (B) in African, Caucasian and East Asian cohorts. Ethnic differences were tested in HOMA-IR (P=0.1770) and HOMA-B (P=0.0008) using subgroup analysis. HOMA-IR, homeostasis model assessment-insulin resistance; HOMA-B, homeostasis model assessment-β-cell function.

Figure S2

Figure S2A.

Figure S2B.

Figure S2. Ethnic distributions of lnHOMA-B relative to lnHOMA-S using liner regression analysis before (A) or after (B) adjustment for age and BMI. The liner functions in African, Caucasian and East Asian are lnHOMA-B=-1.126 lnHOMA-S+4.371, lnHOMA-B=-0.401 lnHOMA-S+4.655 and lnHOMA-B=-0.586 lnHOMA-S+4.346, respectively; after adjustment for age and BMI, the liner functions in African, Caucasian and East Asian are lnHOMA-B=-1.176 lnHOMA-S+4.416, lnHOMA-B=-0.409 lnHOMA-S+5.084 and lnHOMA-B=-0.493 lnHOMA-S+4.055, respectively. HOMA-S, homeostasis model assessment-insulin sensitivity; HOMA-B, homeostasis model assessment-β-cell function.

Figure S3


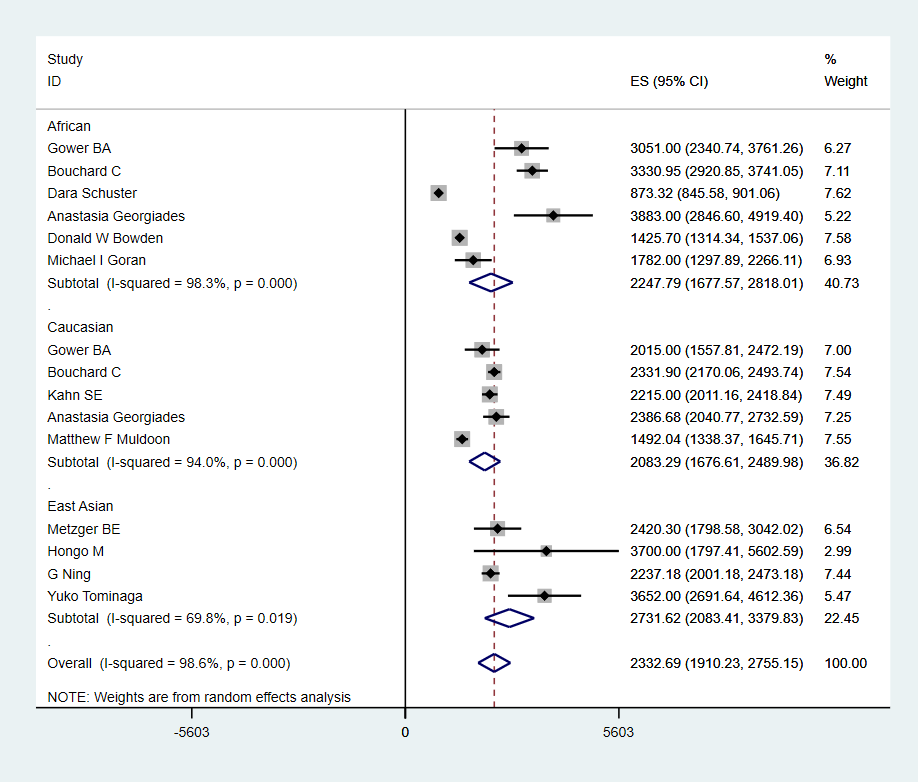


Figure S3. Forest plots of DI in African, Caucasian and East Asian cohorts (mainly NGT) using subgroup analysis. DI was different among Africans, Caucasians and East Asians (P＜0.001). DI: disposition index.

S1: Characteristics of included articles

| races | Year | author | samplesize | age | Age-SD | BMI | BMI-SD | HOMA-IR | HOMA-IR SD | HOMA-B | HOMA-B SD |
| --- | --- | --- | --- | --- | --- | --- | --- | --- | --- | --- | --- |
| African | 2004 | Schuster D | 81 | 41.5 | 4.8 | 31.3 | 3.6 | 2.56 | 1.4 | 391 | 54 |
| African | 2007 | Schuster D | 19 | 49.1 | 7.8 | 32.45 | 6.69 | 2.81 | 2.01 | 252 | 187 |
| African | 2019 | Goff LM | 20 | 32 | 12 | 27 | 3.4 | 0.9259259 |  | 93.1 | 28.4 |
| African | 2013 | Samuel Dagogo-Jack | 217 | 43.5 | 10.3 | 31.2 | 7.36 | 1.93 | 1.8 | 97.2 | 69.6 |
| African | 2005 | Chu A | 231 | 42 | 15.51 | 27.67 | 6.47 | 2.1929825 |  | 140 | 77.54 |
| African | 2011 | Levitt NS | 317 | 32.7 | 7.4 | 24.5 | 5.1 | 1.02 | 0.67 | 77.5 | 48.33 |
| African | 2020 | Hulgan T | 1288 | 39.7 | 9.6 | 28.2 | 7.1 | 2.2 | 1.7 | 186.8 | 123.1 |
| races | Year | Key author's name | samplesize | age | Age-SD | BMI | BMI-SD | HOMA-IR | HOMA-IR SD | HOMA-B | HOMA-B SD |
| Caucasian | 2001 | Yoon C | 71 | 27 | 5.83 | 24.06 | 3.81 | 1.754386 |  | 145.21 | 53.38 |
| Caucasian | 2010 | Ciechanowski K | 50 | 36.7 | 9.2 | 24.4 | 3.7 | 1.3386881 |  | 133 | 68 |
| Caucasian | 2014 | Carolei A | 150 | 36 | 9.88 | 22.9 | 3.43 | 1.63 | 0.84 | 144.47 | 93.87 |
| Caucasian | 2015 | Nakhjavani M | 209 | 52.5 | 11.1 | 28.5 | 4.8 | 1.18 | 0.61 | 141.62 | 128.79 |
| Caucasian | 2016 | Majkowska L | 24 | 35.1 | 5.2 | 21.6 | 1.8 | 1.4 | 0.3 | 132.8 | 24.6 |
| Caucasian | 2017 | Kowalska I | 16 | 24.3 | 4.1 | 22.8 | 3 | 2.6 | 1.2 | 175.1 | 80.7 |
| Caucasian | 2017 | Shestakova MV | 443 | 54.4 | 11 | 28.7 | 4.8 | 2.8 | 1.5 | 94.3 | 30.6 |
| Caucasian | 2018 | Lim S | 43 | 29.9 | 5.9 | 24 | 3.7 | 1.62 | 0.66 | 97.33 | 51.17 |
| Caucasian | 2019 | Kowalska I | 51 | 26.3 | 4.3 | 22.8 | 3.1 | 2.2 | 1.1 | 125 | 70 |
| Caucasian | 2019 | Goff LM | 23 | 36 | 14 | 26.5 | 4.5 | 0.8787346 |  | 97.5 | 37.3 |
| Caucasian | 2013 | Samuel Dagogo-Jack | 159 | 46.6 | 10.5 | 28.8 | 6.81 | 1.91 | 1.5 | 101 | 94.7 |
| Caucasian | 1998 | Conget I | 182 | 36.7 | 12.81 | 25.41 | 4.28 | 2.5477707 |  | 131.63 |  |
| Caucasian | 1999 | Gough SC | 28 | 39.7 | 12.2 | 25.65 | 3.29 | 0.913242 |  | 83.55 | 20.73 |
| Caucasian | 2005 | Chu A | 560 | 47 | 18.1 | 25.88 | 4.95 | 1.8621974 |  | 115 | 66.405 |
| Caucasian | 2008 | Azziz R | 187 | 33.4 | 12.7 | 24.8 | 4.8 | 1.01 | 0.63 | 106.6 | 41.5 |
| Caucasian | 2009 | Atkin SL | 22 | 42.3 | 15.2 | 31.15 | 4.48 | 1.89 | 0.79 | 109.2 |  |
| Caucasian | 2012 | Sun G | 2085 | 42.92 | 12.8 | 26.82 | 5.1 | 1.86 | 2.27 | 132.12 | 551.94 |
| Caucasian | 2015 | Sekikawa A | 151 | 44.8 | 2.8 | 27 | 3.8 | 2.6 | 1.25 | 139.1 | 69.28 |
| Caucasian | 2014 | Rość D | 35 | 29.25 | 5.32 | 22.08 | 2.06 | 1.95 | 0.61 | 154.58 | 51.54 |
| Caucasian | 1997 | Stern MP | 683 | 43.8 | 10.45 | 26.3 | 5.23 | 2.1 | 5.23 | 229.1 | 337.13 |
| Caucasian | 2005 | Baroni MG | 223 | 38.46 | 15.67 | 37.88 | 9.03 | 3.91 | 2.69 | 194.75 | 82.82 |
| Caucasian | 2009 | Ping Wang | 78 | 38.97 | 4.53 | 26.09 | 5.82 | 0.64 | 0.38 | 83.58 | 30.97 |
| Caucasian | 2012 | Gianluca Perseghin | 305 | 37.54 | 9.96 | 27.23 | 7.11 | 1.92 | 1.09 | 153.2 | 63.65 |
| races | Year | author | samplesize | age | Age-SD | BMI | BMI-SD | HOMA-IR | HOMA-IR SD | HOMA-B | HOMA-B SD |
| East Asian | 2001 | Imaizuml T | 119 | 50.6 | 11.2 | 22.5 | 3.1 | 1.5 | 1.5 | 86.1 | 1.5 |
| East Asian | 2003 | Kanauchi K | 238 | 61.6 | 11.2 | 23.2 | 3.1 | 1.62 | 0.96 | 93.7 | 58.5 |
| East Asian | 2004 | Katayama S | 247 | 24.2 | 1.6 | 21.6 | 3.1 | 1.7 | 1 | 146 | 93 |
| East Asian | 2005 | Saruta T | 327 | 49.53 | 8.76 | 23.24 | 2.42 | 1.26 | 0.61 | 62.49 | 29.89 |
| East Asian | 2007 | Shin SJ | 154 | 43.6 | 10.4 | 24.7 | 3.5 | 1.1 | 0.66 | 90.1 | 59.5 |
| East Asian | 2007 | Chan CN | 790 | 35.8 | 8.8 | 23.2 | 2.9 | 1.04 | 0.61 | 127.6 | 64.2 |
| East Asian | 2007 | Koga M | 401 | 51.74 | 5.09 | 22.46 | 2.91 | 0.6726307 |  | 74.23 | 20.62 |
| East Asian | 2009 | Jia W | 542 | 47.72 | 13.33 | 24.94 | 4.14 | 2.26 | 1.55 | 148.83 | 102.51 |
| East Asian | 2009 | Li QF | 153 | 47.77 | 13.45 | 22.99 | 3.34 | 2.66 |  | 156.02 |  |
| East Asian | 2010 | Yanase T | 110 | 24.7 | 1.86 | 22.9 | 3.13 | 1.61 | 1.13 | 119.75 | 67.78 |
| East Asian | 2010 | Kikuchi M | 152 | 58.8 | 6.6 | 23 | 2.9 | 1.73 | 0.86 | 110.2 | 56.2 |
| East Asian | 2010 | Lin X | 1908 | 58.8 | 5.9 | 23.8 | 3.4 | 1.3917884 |  | 126.2 | 44.7 |
| East Asian | 2011 | Ogawa W | 110 | 37.76 | 9.37 | 23.19 | 3.2 | 1.68 | 1.13 | 86.52 | 56.57 |
| East Asian | 2012 | Yang D | 376 | 44.9 | 14.2 | 23.3 | 3.4 | 0.5434783 |  | 67.4 | 0.7 |
| East Asian | 2012 | Yao J | 1183 | 40.44 | 12.4 | 22.66 | 3.16 | 1.52 | 0.88 | 151.49 | 335.1 |
| East Asian | 2012 | Tobe K | 763 | 72.5 | 9 | 22.7 | 3.3 | 1.24 | 0.78 | 60.8 | 41.2 |
| East Asian | 2012 | Feng B | 189 | 65.07 | 8.93 | 24.59 | 3.07 | 1.52 | 0.87 | 99.77 | 61.45 |
| East Asian | 2012 | Feng B | 138 | 64.7 | 8.92 | 24.57 | 3.15 | 1.42 | 0.76 | 98.19 | 62.73 |
| East Asian | 2012 | Li WH | 295 | 33.27 | 10.39 | 23.95 | 3.52 | 1.56 | 1.05 | 125.73 | 87.65 |
| East Asian | 2012 | Tong N | 123 | 53.6 | 10.1 | 23.93 | 2.89 | 1.45 |  | 148.1 | 136.55 |
| East Asian | 2013 | Pei D | 19 | 54.1 | 8.8 | 26.3 | 3.2 | 0.63 |  | 50.12 |  |
| East Asian | 2013 | Hu Z | 88 | 58.81 | 6.69 | 24.96 | 3.69 | 1.7 | 0.99 | 92.22 | 41.67 |
| East Asian | 2013 | Liu L | 2001 | 43.5 | 11.6 | 23.4 | 3.7 | 2.4 | 1.3 | 113.4 | 65.2 |
| East Asian | 2014 | Chen B | 331 | 47.73 | 8.49 | 24.56 | 3.15 | 2.94 | 1.36 | 126.06 | 67.95 |
| East Asian | 2014 | Ando E | 240 | 57.21 | 16.29 |  |  | 1.27 | 0.92 | 103.58 | 111.6 |
| East Asian | 2015 | Cho YM | 192 | 53 | 6.99 | 23.96 | 2.9 | 1.24 | 0.71 | 100.42 | 54.99 |
| East Asian | 2015 | Nakayama T | 352 | 49.89 | 8.35 | 22.68 | 2.86 | 1.26 | 0.66 | 66.56 | 37.01 |
| East Asian | 2015 | Li XS | 90 | 48.3 | 12.23 | 21.5 | 2.01 | 1.95 | 0.67 | 109 | 41.5 |
| East Asian | 2016 | Chen ZJ | 67 | 28.55 | 4.15 | 25.87 | 4.61 | 4.1 | 2.62 | 156.22 | 94.78 |
| East Asian | 2017 | Cho NH | 5703 | 51.4 | 8.7 | 24.3 | 3 | 1.3 | 1.88 | 127 | 2 |
| East Asian | 2016 | Nishimura M | 83 | 34 | 5.7 | 22.5 | 2.4 | 1.2 | 0.8 | 71.3 | 37.8 |
| East Asian | 2016 | Sun K | 23 | 49.2 | 10.6 | 25.6 | 4.7 | 2.49 | 0.74 | 156.33 |  |
| East Asian | 2016 | Li P | 69 | 50 | 11 | 24 | 3 | 1.5 | 1.1 | 110 | 156 |
| East Asian | 2016 | Li Y | 200 | 48.73 | 12.06 | 25.09 | 3.44 | 3.25 |  | 100.2 |  |
| East Asian | 2017 | Qiao H | 976 | 42.9 | 11.7 | 23.3 | 3.3 | 1.7 | 1 | 118 | 225.2 |
| East Asian | 2017 | Zhang Y | 376 | 48.5 | 11.8 | 23.6 | 3.3 | 0.73 | 0.5 | 70 | 29.9 |
| East Asian | 2018 | Lim S | 43 | 30 | 5.2 | 24.1 | 3.2 | 1.24 | 0.46 | 75.31 | 37.64 |
| East Asian | 2018 | Zheng H | 52 | 54.73 | 11.9 | 22.76 | 3.61 | 1.38 | 0.88 | 71.03 | 41.79 |
| East Asian | 2019 | Hong Q | 1000 | 42 | 19 | 23.14 | 4.8 | 1.51 | 1.17 | 104.84 | 78.74 |
| East Asian | 2020 | Yang X | 138 | 27.9 | 8.2 | 32.8 | 4.4 | 5.5 | 3 | 327 | 226 |
| East Asian | 2020 | Ma RC | 80 | 49.9 | 4.45 | 24.6 | 3.9 | 0.78 | 0.51 | 89.9 | 43.6 |
| East Asian | 2006 | Chan JC | 431 | 36 | 13 | 23.6 | 4 | 1.7 | 1.06 | 123 | 90.03 |
| East Asian | 2009 | Jia WP | 84 | 57.64 | 9.54 | 25.74 | 3.82 | 2.27 | 1.95 | 131.06 | 99.35 |
| East Asian | 2010 | Ji L | 1005 | 58 | 9 | 25 | 3.3 | 1.7 | 0.91 | 83.43 | 40.16 |
| East Asian | 2010 | Wang WQ | 65 | 28.1 | 3.4 | 20.6 | 1.7 | 1.46 | 0.38 | 114 | 65.66 |
| East Asian | 2010 | Ng MC | 583 | 41.4 | 10.5 | 22.9 | 3.3 | 1.51 | 0.86 | 110.39 | 70.05 |
| East Asian | 2010 | Lee YJ | 994 | 60.7 | 16.9 | 23.7 | 3.6 | 2.07 | 0.82 | 98.7 | 42.39 |
| East Asian | 2011 | Yokoyama H | 371 | 47.98 | 5.97 | 22.86 | 2.78 | 1.1 | 1.04 | 63.02 | 44.83 |
| East Asian | 2012 | Liu W | 123 | 27.43 | 4.41 | 22.99 | 4.82 | 1.87 | 1.42 | 155.26 | 155.53 |
| East Asian | 2014 | Yang W | 4663 | 49.7 | 8.9 | 23.07 | 2.6 | 1.47 | 0.59 | 90.96 | 47.9 |
| East Asian | 2013 | Liu L | 90 | 48.26 | 9.98 | 23.38 | 2.95 | 1.57 | 0.74 | 78.87 | 50.22 |
| East Asian | 2013 | Zhang ZL | 1382 | 37.13 | 12.35 | 21.83 | 2.6 | 1.33 | 0.6 | 135.78 | 72.25 |
| East Asian | 2014 | Yu X | 46 | 48 | 10 | 26.3 | 4.84 | 2.98 | 0.23 | 216.2 | 28.49 |
| East Asian | 2015 | Sekikawa A | 93 | 44.8 | 2.8 | 22.4 | 2.9 | 2.1 | 0.97 | 101.9 | 57.3 |
| East Asian | 2014 | Gu Y | 2906 | 69.9 | 6.5 | 23.4 | 3.2 | 1.37 | 0.74 | 81.27 | 48.06 |
| East Asian | 2014 | Tong N | 590 | 49.19 | 6.73 | 23.62 | 2.93 | 1.28 | 0.65 | 90.51 | 44.44 |
| East Asian | 2010 | Jia W | 3412 | 50.1 | 14.27 | 23.46 | 3.25 | 1.39 | 0.72 | 96.43 | 54.29 |
| East Asian | 2014 | Aizawa T | 604 | 53 | 8.92 | 23.44 | 2.6 | 0.8333333 |  | 44.54 | 21.62 |
| East Asian | 2015 | Takeda J | 702 | 59.11 | 9.84 | 22.93 | 3.17 | 1.14 | 0.69 | 69.3 | 37.95 |
| East Asian | 2015 | Yu X | 50 | 53.34 | 7.56 | 24.45 | 2.06 | 1.57 | 0.85 | 37.71 | 27.03 |
| East Asian | 2016 | Wang G | 1712 | 43.2 | 11.5 | 25.07 | 3.51 | 3.03 | 1.62 | 126.56 | 65.38 |
| East Asian | 2016 | Jia W | 194 | 48 | 14 | 23.08 | 2.7 | 2.11 | 1.17 | 138.97 | 90.43 |
| East Asian | 2016 | Yang T | 2514 | 55.1 | 9 | 23.6 | 3 | 2.42 | 1.1 | 114.17 | 53.11 |
| East Asian | 2017 | Li Y | 427 | 50.62 | 6.55 | 23.16 | 2.86 | 1.4 | 0.79 | 96.4 | 50.34 |
| East Asian | 2017 | Choi SH | 1744 | 35 | 5.4 | 22.8 | 3.3 | 2.04 | 0.67 | 139.81 | 47.04 |
| East Asian | 2017 | Nakagawa H | 575 | 42.3 | 6.8 | 23.1 | 3.1 | 1.12 | 0.56 | 64.2 | 33.37 |
| East Asian | 2017 | Deng H | 39 | 47.38 | 12.62 | 22.82 | 2.87 | 1.16 | 0.24 | 64.72 | 17.11 |
| East Asian | 2017 | Yu X | 198 | 53.18 | 8.61 | 24.66 | 2.83 | 1.52 | 0.75 | 86.97 | 45.48 |
| East Asian | 2018 | Yang T | 1275 | 55.81 | 9.26 | 23.39 | 3.42 | 2.45 | 1.18 | 115.55 | 56.9 |
| East Asian | 2018 | Li Q | 232 | 60.8 | 11.3 | 22.8 | 2.7 | 0.7 | 0.3 | 95.59 | 107.87 |
| East Asian | 2019 | Dong GH | 13783 | 43.9 | 13.4 |  |  | 1.94 | 1.04 | 114.18 | 63.83 |
| East Asian | 2019 | Liu M | 81 | 31.32 | 7.95 | 24.69 | 4.56 | 2.04 |  | 116.86 |  |
| East Asian | 2008 | Seino Y | 556 | 43.92 | 10.96 | 23.47 | 2.62 | 1.14 | 0.69 | 71.61 | 42.67 |
| East Asian | 2009 | Luo M | 723 | 35.68 | 18.2 | 24.42 | 9.14 | 0.6456612 |  | 107.15 |  |
| East Asian | 2019 | Masami Murakami | 575 | 23.7 | 1.7 | 21.1 | 2.5 | 1.47 | 0.92 | 86.43 | 40.95 |
| East Asian | 2008 | Guang Ning | 721 | 59.7 | 12.4 | 24.1 | 3.2 | 1.01 | 10.21 | 52.4 | 10.69 |
| East Asian | 2013 | Susumu Seino | 43 | 30.2 | 8.8 | 20.9 | 2.1 | 1.14 | 0.41 | 92.1 | 36.4 |
| East Asian | 2014 | Tomohiro Nakayama | 341 | 50.3 | 8.2 | 22.6 | 2.8 | 1.2 | 0.7 | 65.7 | 36.7 |
